# Engineering self-organising helium bubble lattices in tungsten

**DOI:** 10.1038/s41598-017-07711-w

**Published:** 2017-08-10

**Authors:** R. W. Harrison, G. Greaves, J. A. Hinks, S. E. Donnelly

**Affiliations:** School of Computing and Engineering, University of Huddersfield, Huddersfield, HD1 3DH UK

## Abstract

The self-organisation of void and gas bubbles in solids into superlattices is an intriguing nanoscale phenomenon. Despite the discovery of these lattices 45 years ago, the atomistics behind the ordering mechanisms responsible for the formation of these nanostructures are yet to be fully elucidated. Here we report on the direct observation via transmission electron microscopy of the formation of bubble lattices under He ion bombardment. By careful control of the irradiation conditions, it has been possible to engineer the bubble size and spacing of the superlattice leading to important conclusions about the significance of vacancy supply in determining the physical characteristics of the system. Furthermore, no bubble lattice alignment was observed in the <111> directions pointing to a key driving mechanism for the formation of these ordered nanostructures being the two-dimensional diffusion of self-interstitial atoms.

## Introduction

The formation of void and gas bubble superlattices is an intriguing nanoscale phenomenon^1–7^. Authors have previously reported the formation of void and gas bubble superlattices in tungsten under irradiation conditions^3–8^. Tungsten is regarded as a primary candidate for use as a plasma facing material in the divertor of the International Thermonuclear Experimental Reactor (ITER) and the DEMOnstration (DEMO) fusion reactors^9^. During its operational lifetime, the divertor will be subject to bombardment from He atoms ﻿an﻿d 14.1 MeV neutrons resulting from D-T fusion reactions leading to a mixture of displacement damage and He implantation^10^. The conditions needed (e.g. radiation damage dose and temperature) for void and gas bubble superlattice formation^8, 11, 12^ are likely to be experienced by the divertor in-service. Therefore, thorough characterisation and understanding of these superlattices and the fundamental mechanisms behind their creation are crucial.

Void superlattices (henceforth referred to as “lattices” for brevity) have been observed in neutron-irradiated W (where the He concentration is <1 appm) by Sikka and Moteff^12^ where they irradiated up to a fluence of ~10^22^ n.cm^–2^ in the Experimental Breeder Reactor (EBR-II) at 823 K. They reported an average void diameter of 3 nm and a void-lattice spacing of 19.5 nm. Tanno *et al*.^11, 13^ also reported the formation of a void lattice in fast-neutron-irradiated W at 1023 K at a similar fluence of ~10^22^ n.cm^–2^ (~1.5 displacements per atom (DPA)), finding similar void-lattice parameters to those reported by Sikka and Moteff^12^ with a void diameter of 5 nm and void-lattice spacing of 20 nm. The slightly-larger void diameter reported by Tanno *et al*.^11, 13^ may have been due to the higher temperature of their experiments compared to those of Sikka and Moteff^12^. Helium bubble lattices in W have been reported by Johnson and Mazey^8^ using 50 keV He ions up to a fluence of 1.5 × 10^17^ ions.cm^–2^. The bubble diameter reported by Johnson and Mazey^8^ was ~2 nm with a bubble-lattice constant of 6.2 ± 0.2 nm found from both transmission electron microscopy (TEM﻿) image an﻿d selected area diffraction pattern (SADP) analysis.

**Figure 1:**
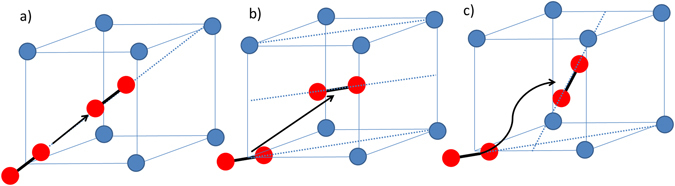
Schematic showing: (**a**) Diffusion of a <111> crowdion in the close-packed <111> direction in the BCC structure as described by the 1-D SIA model; (**b**) diffusion of a <110> dumbbell in the (110) plane as described by the 2-D SIA diffusion model; and (**c**) forbidden rotation of a <110> dumbbell onto the (011) plane which would give rise to 3-D SIA diffusion.

These void and bubble lattices observed in both body-centred cubic (BCC) and face-centred cubic (FCC) metals have received much attention in previous decades in attempts to understand their formation mechanism(s)^1–3, 5, 8, 11–22^. Models proposed have been based on elastic interaction between cavities^23^, isomorphic decomposition^24^, phase instability, interstitial loop punching^25^, 1-D self-interstitial atom (SIA) diffusion^15^ and/or 2-D SIA diffusion^16^. In the case of mechanisms based on anisotropic SIA migration^15, 16^, cavities nucleate randomly and those that are serendipitously aligned with one another along the direction or plane of SIA migration will experience a shadowing effect from anisotropically migrating SIAs (we use “cavity” to refer to either bubbles or voids without distinction). However, the precise nature of the formation mechanism of these cavity lattices is still, after several decades, a subject of much debate^2, 3, 15, 18, 26, 27^. In BCC metals, a 1-D SIA diffusion mechanism may occur along the close packed <111> directions (shown in Fig. 1a). However, void lattices observed in neutron-irradiated Mo did not show alignment along these directions putting this model into question^28, 29^. The 2-D SIA diffusion mechanism proposed by Evans^16, 18^ involved the diffusion of <110> dumbbells along the {110} planes in BCC metals (shown in Fig. 1b and c) which is consistent with ordering of both void and bubble lattices on {110} planes. However, this mechanism assumes that the <110> dumbbell does not rotate onto another {110} plane (giving 3-D mobility) due to an energy penalty (Fig. 1c). Such an energy penalty is supported by experimental observation of electron-irradiated Mo at 5 K^30^. Evans^31^ proposed that the planar 2-D migrating defect must be a di-interstitial as simulations predicted cavity lattices at much lower DPAs than were observed experimentally. Thus, it was concluded that the di-interstitial which is present in a much lower concentration than the mono-interstitial must be responsible.

The mechanism proposed by Evans^18^ based on the 2-D diffusion of <110> type dumbbells is, however, inconsistent with recent density functional theory (DFT) simulations in which the <110> dumbbell was found to be stable only in Fe and the <111> crowdion was found to be the most stable SIA configuration for other BCC metals including W^32^. Recent molecular dynamics (MD) simulations^33^ based on the <111> crowdion configuration predicted from DFT simulations^32^ have been used to examine the dynamics of these defects at elevated temperatures. The simulations indicated that for temperatures up to 400 K, motion of the SIAs was restricted to 1-D with the SIAs performing stochastic longitudinal motion on the <111> axes with a migration energy of 0.018 eV. At temperatures above 400 K, the crowdion was able to rotate into one of the three other equivalent <111> configurations with an activation energy of ~0.4 eV. Rotation between the <111> axes involves passing through a <110> type configuration which has been linked to a slowing of the migration rate^32^. Heinish and Singh^27^ examined the 1-D migration of crowdion clusters, with an occasional Burgers vector change, in the context of void alignments using kinetic Monte Carlo simulations in the FCC structure. The authors found that, for near-perfect void lattices, the mean free path (*L*) of the SIA moving in 1-D before it rotated had to be around three times larger than the bubble nearest-neighbour mean distance for the void lattice to form. Amino *et al*.^26^ used high-voltage (1 MeV) TEM at a low temperature (16 K) coupled with object kinetic Monte Carlo simulations to explore the growth of interstitial clusters in W and showed that the growth rate of these clusters follows rate kinetics as predicted by the 1-D SIA diffusion model. This is to be expected to occur at this temperature as, according to MD simulations, the rotation of the <111> crowdion is not possible below ~400 K^33^.

Reviews of void and gas bubble lattices by Krishan^14^ and by Johnson and Mazey^8^ noted the differences in lattice parameters between void lattices and bubble lattices with the former being generally 5–10 times larger. With regard to void-lattice spacings in neutron-irradiated metals, Hähner and Frank^34^ concluded that the void-lattice parameter is related to the void density in the random phase before anisotropic SIA migration results in ordering. It is important to develop a fundamental understanding of the effects of He on these superstructures in W as He bubble and void lattices are produced in temperature regimes (0.2–0.4 *T*_m_) and irradiation conditions expected across the divertor structure in the ITER and DEMO reactors. Using TEM with *in situ* ion irradiation, it is possible to vary the amount of He injected into a thin film simply by altering the energy of the incident He beam. This paper reports on the use of the *in situ* TEM technique to explore the effect of varying He concentration/damage on the He bubble-lattice parameters.

## Results and Discussions

### Characterisation of He bubble-lattice parameters

**Figure 2:**
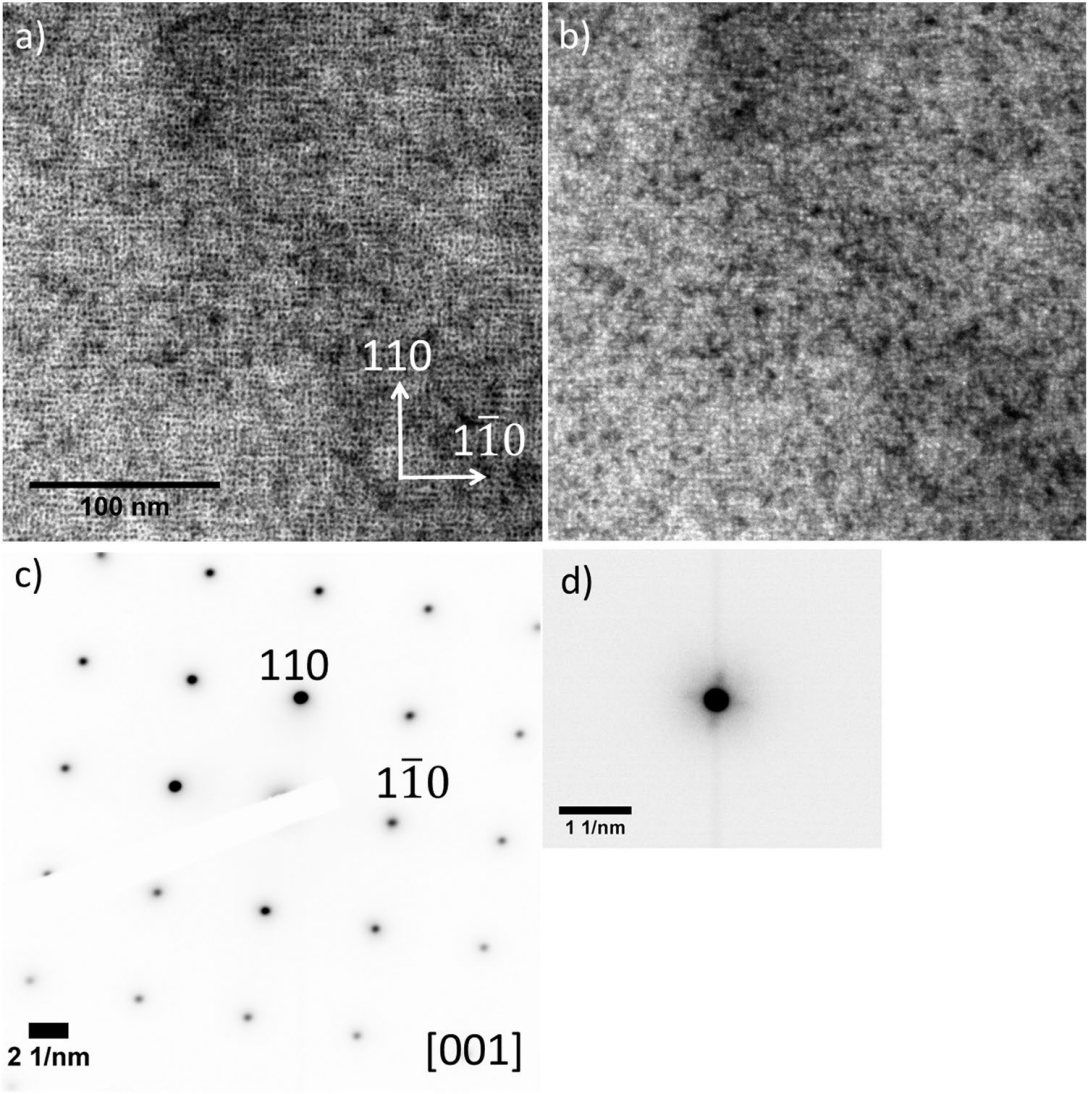
W sample irradiated with 15 keV He ions at 773 K up to 1.1 × 10^17^ ions.cm^−2^ to 3.0 DPA: (**a**) BF-TEM image of a He bubble lattice taken at 1.2 μm overfocus; (**b**) BF-TEM image of a He bubble lattice taken at 1.2 μm underfocus; (**c**) SADP of the region in (a); and (**d**) DP of the He bubble lattice around the 000 spot. The scale marker in (a) applies to both micrographs.

Bubble lattices were formed at all He ion energies studied. Examples are given in Fig. 2a and b showing bright field (BF) TEM images in under and overfocus of a sample irradiated at 773 K with 15 keV He ions to a fluence of 1.1 × 10^17^ ions.cm^−2^ (He concentration of 1.2 × 10^5^ appm and dose of 3.0 DPA with He-appm/DPA ratio ~40,000). The images were taken close to the [001] W zone axis as confirmed by the SADP in Fig. 2c and ordering of bubbles along the {110} planes is clear. Measurement of the *d*_bubble_(110) spacings from the image yielded 3.1 ± 0.4 nm corresponding to a *a*_bubble_ of 4.4 ± 0.4 nm. These values are in good agreement with the lattice constants measured from the SADP in Fig. 2d where the *d*_bubble_(110) was found to be 3.2 ± 0.2 nm and *a*_bubble_ = 4.6 ± 0.2 nm corresponding to a density of ~2.05 × 10^19^ bubbles.cm^–3^ (calculated from the measured lattice parameter and the assumption that the bubble lattice adopts the BCC structure).

**Figure 3:**
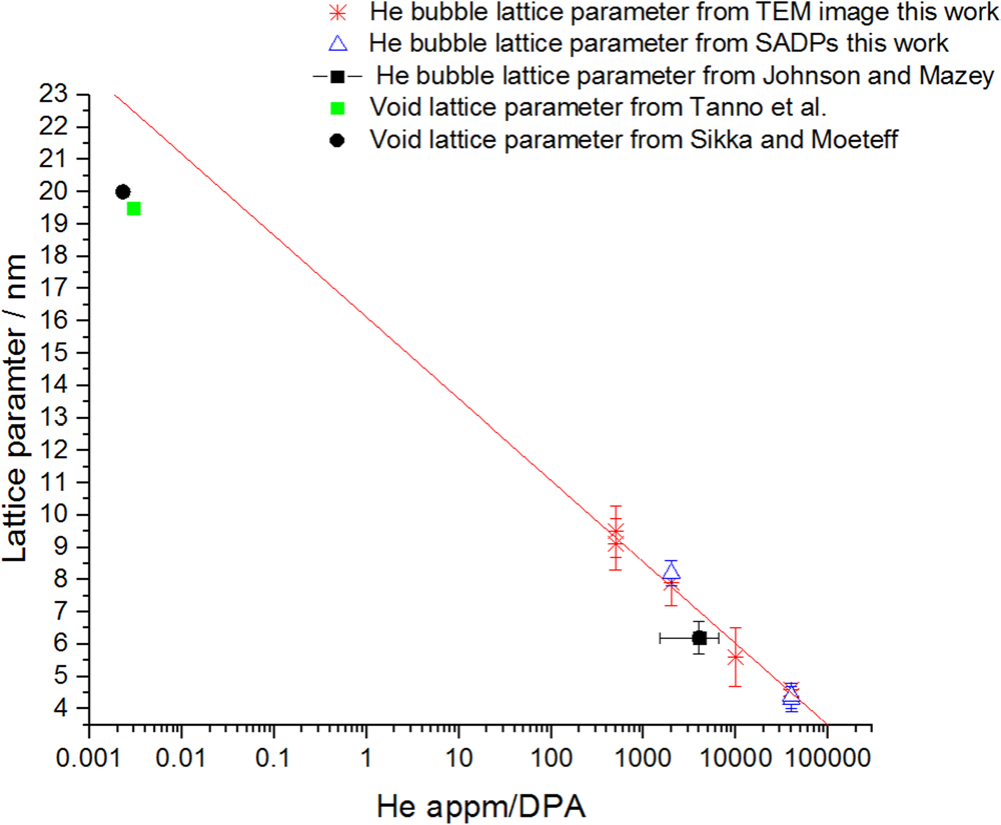
He bubble-lattice parameter in He ion-irradiated W samples as a function of the log of the He-appm/DPA ratio with previous work on He ion irradiation^8^ and void-lattice spacings from neutron-irradiated W^12, 13^. (Line of best fit shows linear fit of data reported in this work only.)

**Figure 4:**
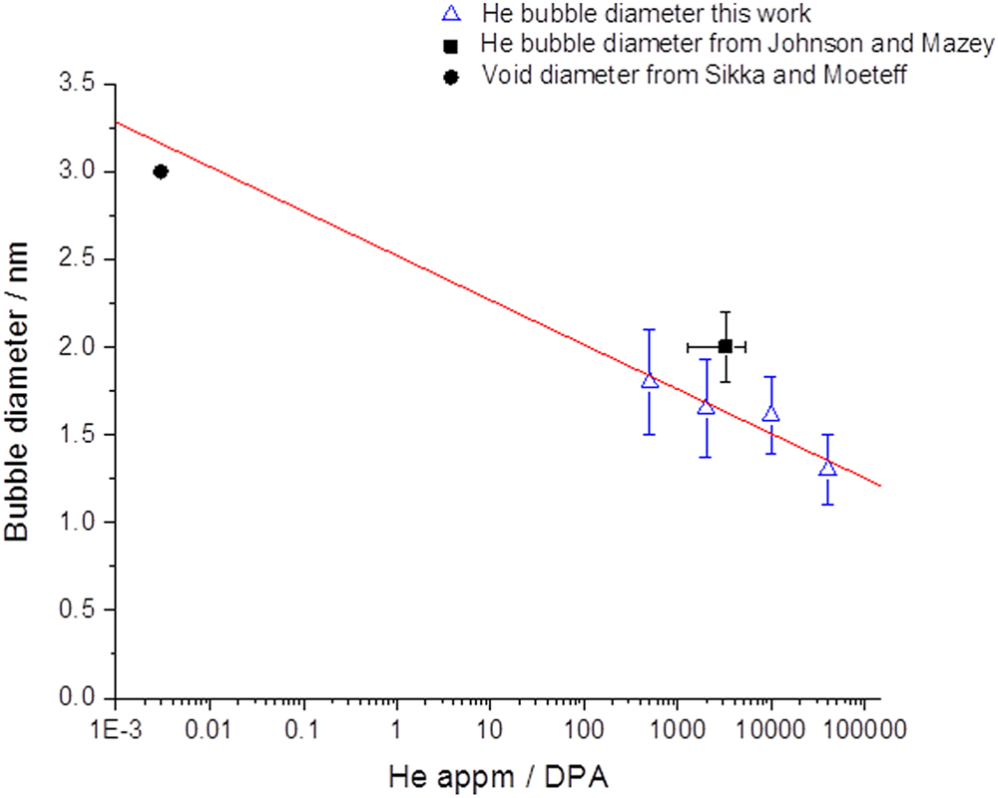
He bubble diameter in He ion-irradiated W samples as a function of the log of the He-appm ratio with previous work on He ion irradiation^8^ and void diameters from neutron-irradiated W^12^. (Line of best fit shows linear fit of data reported in this work only.)

Figure 3 shows a plot of the log of He-appm/DPA ratio versus *a*_bubble_ measured from the images and SADPs. This reveals a linear trend showing that as the He appm/DPA decreases (i.e. as He concentration decreases) the lattice parameter increases (BF-TEM images of bubble lattices for each irradiation can be found in the Supplementary Material). The value of *a*_bubble_ reported by Johnson and Mazey^8^ using 50 keV He ion irradiation of W at 773 K falls in line with the trend observed in this work as can be seen in Fig. 3. In that work^8^, bulk samples of W were He ion irradiated and then back-thinned from the non-irradiated side to a thickness of 50–100 nm thus effectively selecting an amount of implanted He as well as total DPA. The corresponding range in He-appm/DPA ratio is represented by the x-error bar in Fig. 3 as calculated from SRIM. Void-lattice parameter measurements from neutron-irradiated W from Sikka and Moteff^12^ and Tanno *et al*.^11, 13^ also fall onto the exponential trend observed in the current work. For the neutron-irradiated cases, the He concentration due to transmutation reactions and DPA has been estimated using the FISPACT-II^35^ neutron-transport code. Figure 4 shows that bubble diameters also followed the same trend demonstrating a decrease in size with increasing He concentration. This indicates that there is a direct relationship between He bubble-lattice parameter and He appm/DPA ratio which has not previously been explored in a systematic manner as far as the authors are aware.

### Effect of He concentration on bubble-lattice parameters

The mobility of defects in neutron- and ion-irradiated W has been the subject of many studies into the mechanisms which drive radiation damage recovery^11, 36–41^. The first stage of recovery is the diffusion of free interstitials which occurs below 100 K^39^. Above 100 K, interstitials can escape from traps and annihilate at sinks (e.g. vacancy-type defects, grain boundaries and surfaces). Monovacancy migration in W has an activation energy of 1.7 eV^39, 42^ and becomes important at temperatures above 620 K. In this work, there is an excess of vacancies created compared to He atoms implanted with displacements per ion impact ≥3. Thus it is more likely a migrating He atom will encounter a vacancy rather than another He atom. Gonzalez and Iglesias^43^ calculated migration energies of He and He-vacancy complexes in W and reported that the migration energy of a monovacancy increases from ~1.8 to 4.83 eV with the addition of a single He atom. At higher He appm/DPA ratios, there will be a greater likelihood of less-mobile He-vacancy complexes compared to monovacancies and vacancy clusters thus preventing these He-vacancy complexes from migrating and being absorbed by other He-vacancy complexes. This will give rise to a higher nucleation density of small bubbles and He-vacancy complexes during the random nucleation phase and thus a smaller inter-bubble spacing (*a*_bubble_) when the bubble lattice forms during ordering due to anisotropically migrating SIAs.

In the neutron irradiation case, relatively-little He is present (<1 appm He). At temperatures where vacancies are mobile and can be captured by voids or otherwise lost to sinks, a lower density of larger voids would be generated in the random phase which results in larger void-lattice spacings compared with the bubble-lattice spacings in this work. Voids in neutron-irradiated W have been reported to be around 3 nm (ref. 12) and 5 nm (ref. 13) in diameter whereas bubble diameters in the present work were found to be smaller at ~1.4 nm (similar to the value of ~2 nm reported by Johnson and Mazey^8^ for He ion-irradiated W). This difference of around a factor of two is consistent with the arguments put forward here in which the pinning of vacancies is less likely in neutron-irradiated W resulting in a lower nucleation density and greater cavity size; whereas the chance of vacancies becoming pinned is increased in the presence of He leading to a higher density of bubbles with smaller diameters under He irradiation.

### On the mechanism of bubble lattice formation

**Figure 5:**
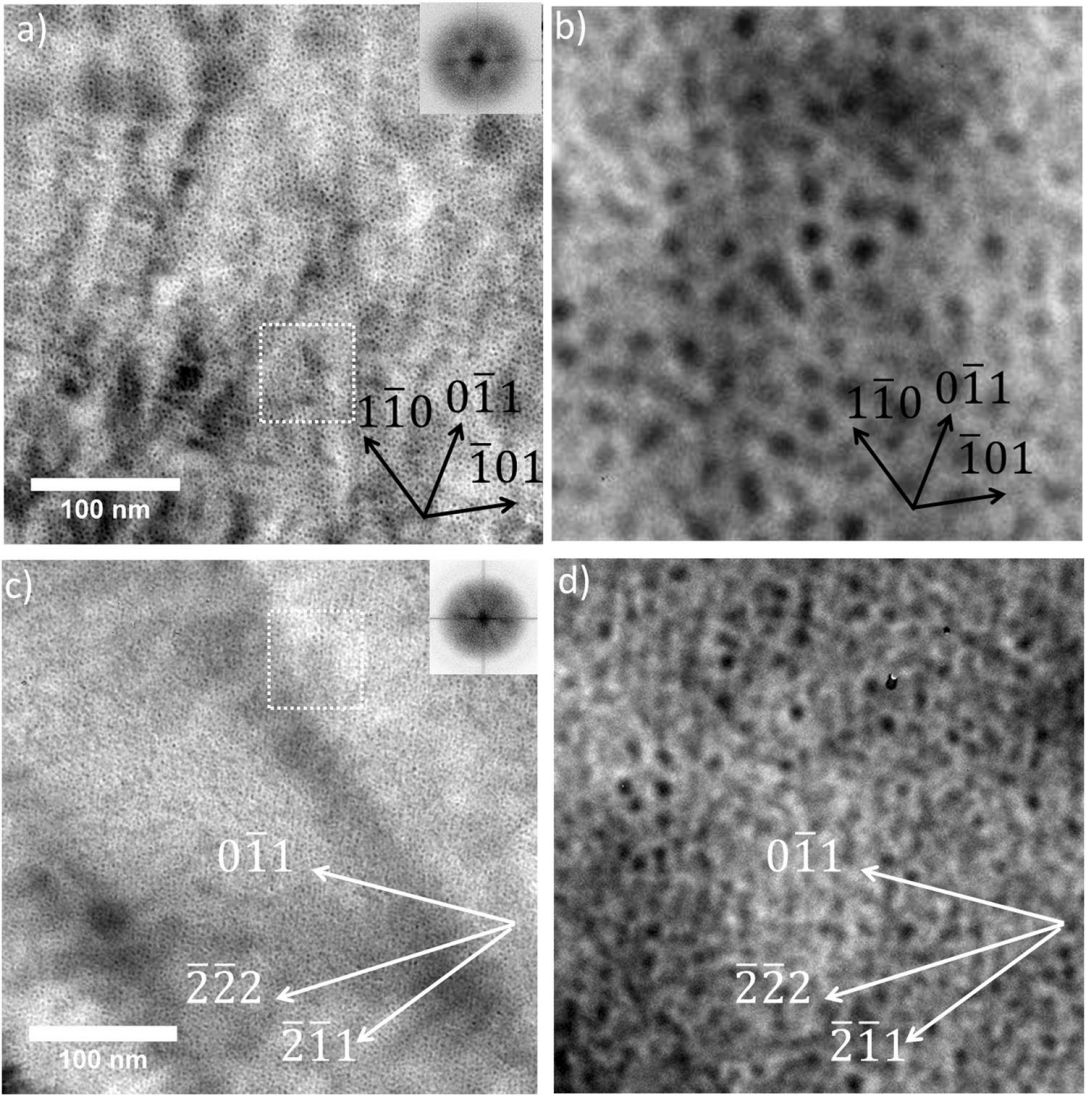
W sample irradiated with 15 keV He ions at 773 K up to 1.1 × 10^17^ ions.cm^−2^ to 3.0 DPA: (**a**) BF-TEM image of a He bubble lattice close to the [111] zone axis with inset showing the corresponding FFT; (**b**) enlarged region of white dashed box in (a); (**c**) BF-TEM image of He bubble lattice close to the [011] zone axis with inset showing the corresponding FFT; and (**d**) enlarged region of white dashed box in (c).

Figures 5a–d show BF-TEM images in overfocus conditions of a W sample irradiated at 773 K with 15 keV He ions to a fluence of 1.1 × 10^17^ ions.cm^−2^ (He concentration of 1.2 × 10^5^ appm and dose of 3.0 DPA with a He-appm/DPA ratio ~40,000). Figures 5a and b show BF-TEM images of the sample imaged close to the [111] zone axis; it can be seen from the image and the FFT inset in Fig. 5a that there is alignment of bubbles on all {110} type planes (lobes on FFT are equally separated by ~60° matching well with the [111] direction). However, Figs. 5c and d show BF-TEM images of the same sample imaged close to the [011] zone axis. Alignment can be seen in the $(0\bar{1}1)$ plane and [$0\bar{1}1$] direction from the FFT inset into Fig. 5c. The additional lobes in the FFT arose from directionality in the $(\bar{2}\bar{1}1)$ planes as these indexed well with the (110) FFT lobes as they were separated by an angle of ~55°. However, no directionality is observed in the <111> directions from Figs. 5c and d. The 1-D SIA migration mechanism would result in bubble ordering in the <111> directions and within the {111} planes. In the absence of this observation, our results agree with previous work^28, 29^ where no void alignment in neutron-irradiated Mo in <111> directions was observed. This indicates the 2-D SIA mechanism is responsible for this phenomenon contrary to recent simulation^32, 44^ and experimental work^26^ supporting the 1-D SIA migration mechanism.

## Conclusion

He bubble lattices in W were observed for all He ion irradiations at 773 K. The main conclusions arising from this work are:

1. Observation of a novel trend between bubble-lattice parameter and He concentration/damage ratio. This is attributed to higher He concentrations inhibiting the mobility of vacancies and thus producing a high nucleation density of small closer-spaced bubbles in the random phase before ordering occurs.
2. The formation mechanism of these bubble lattices results from 2-D SIA migration as no bubble ordering was observed in the <111> directions which would result from a 1-D SIA migration mechanism in BCC materials.
3. Bubble lattices readily form under these irradiation conditions (He concentration, temperature and DPA) which are anticipated under in-service conditions in ITER. This has great importance to further work aimed at elucidating the potential materials challenges and problems, such as enhanced degredation of mechanical properties, that could result from an ordered, rather than random, arrangement of bubbles.

## Methods

Samples of W were prepared by punching 3 mm discs from foil (Alfa-Aesar, 99.95 wt.%, main impurity C 0.003 wt.%). The discs were then annealed at 1673 K for two hours under vacuum (0.1 Pa) to remove any pre-existing effects from cold working. After annealing, the discs were electro-chemical jet polished at room temperature with 0.5 wt.% NaOH solution using a Tenupol-5. Samples were subsequently washed in three separate baths of CH_3_OH to remove any residue from the electrolyte.

*In situ* ion irradiation was performed using the Microscopes and Ion Accelerators for Materials Investigations (MIAMI-1) facility^45^. This consists of a JEOL JEM-2000FX TEM operating at 200 kV coupled to an ion accelerator operating at energies of up to 100 keV. The ion beam is oriented at 30° to the electron beam. In these irradiations the specimens were oriented normal to the electron beam and were heated using a Gatan 652 double-tilt heating holder. Post-irradiation examination was also performed with a JEOL JEM-3010 TEM operated at 300 kV. Helium bubbles were imaged using Fresnel contrast where small cavities appear light in underfocus and dark in overfocus. The cavities are assumed to be bubbles and not voids due to the high concentrations of implanted helium in these experiments. MoO_3_ crystals were used to calibrate the image rotation between the diffraction patterns and micrographs. A clockwise rotation of the diffraction pattern with respect to the image was found with angles between 4° and 6° when using magnifications of 20k and 40k, respectively, for a camera length of 20 cm.

Helium bubble-lattice constants were determined by measuring the distance between the planes directly from images or from reflections of the He bubble lattice in the SADPs. Where bubble spacings were determined from an image, a measurement of 30 bubble spacings was taken using ImageJ^46^. The errors in the measurements of bubble spacings taken directly from the image are given as the standard deviation of the 30 measurements and errors from SADPs are estimated as 5% due to the imprecision in determination of the centre of the diffraction spots. SADPs were indexed by matching with reference patterns using SingleCrystal software (CrystalMaker version 2.2.9) and bubble-lattice parameters (*a*_bubble_) were calculated from these indexed patterns and measured *d*_bubble_ spacings using Equation 1.

$${{a}}_{{\rm{b}}{\rm{u}}{\rm{b}}{\rm{b}}{\rm{l}}{\rm{e}}}{\rm{=}}{{d}}_{{\rm{b}}{\rm{u}}{\rm{b}}{\rm{b}}{\rm{l}}{\rm{e}}}\sqrt{{{h}}^{{2}}{\rm{+}}{{k}}^{{2}}{\rm{+}}{{l}}^{{2}}}$$

As the He bubble lattice adopts the BCC structure in W, bubble densities (bubbles.cm^−3^) were calculated from these lattice parameters.

Damage and He concentrations were calculated using the *Stopping and Range of Ions in Matter* (SRIM-2013) Monte Carlo computer code^47^. The damage in units of DPA was calculated via the method proposed by Stoller *et al*.^48^ using the ‘Quick’ Kinchin-Pease option of SRIM for 5000 ions with a displacement energy of 90 eV^49^ and binding energies set to 0 eV. Helium concentrations were determined from SRIM calculations using the ‘Quick’ method to 99,999 ions to ensure good statistics with the foil thickness set to 50 nm. Samples were irradiated to 3.0 DPA (averaged across the TEM foil) using 15, 30, 60 and 85 keV He ions resulting in implanted He concentrations of 1.2 × 10^5^, 3.0 × 10^4^, 6.5 × 10^3^ and 1.6 × 10^3^ appm (averaged across the TEM foil), respectively. Plots of damage (in DPA) and He concentration (in appm) as a function of depth from SRIM are in the supplementary information illustrating the relatively-flat damage profiles achieved. Helium concentration was generally peaked in the centre of the foil. However, He is very mobile in metals and point defects (interstitials and vacancies) will be mobile at this irradiation temperature leading to a fairly homogeneous distribution of He and damage throughout the foil. It is assumed cavities observed in all irradiations are bubbles as the high concentration of cavities will provide the dominant sink for further He trapping. Previous studies have also determined He bubble pressures and densities of similar-sized bubbles produced from irradiations at He ion energies^50, 51^ or He concentrations (appm)^52^ on the same order of magnitude as those reported here. In order to facilitate comparison with previous He ion irradiation and neutron work, it is important to account for both He-appm and DPA. He-appm/DPA ratios of 40,000, 9900, 2000 and 500 were achieved for He ion energies of 15, 30, 60 or 85 keV, respectively. The flux was set for each He ion energy so as to keep the DPA rate constant at 10^−3^ DPA.s^−1^ and all samples were irradiated at 773 K as this is 0.2 *T*_m_ where bubble lattices have been observed previously in the literature^8, 14, 53^.

## Electronic supplementary material

Supplementary material

## References

[1] Evans JH (1971). Observations of a Regular Void Array in High Purity Molybdenum irradiated with 2 MeV Nitrogen Ions. Nature.

[2] Dubinko VI, Tur AV, Turkin AA, Yanovskij VV (1989). “A mechanism of formation and properties of the void lattice in metals under irradiation. J. Nucl. Mater..

[3] Evans, J. H. Simulations of the effects of 1-d interstitial diffusion on void lattice formation during irradiation. *Philos. Mag*. **85**, August 2014, 1177–1190 (2005).

[4] Johnson PB, Mazey DJ (1979). Helium gas-bubble superlattice in copper and nickel. Nature..

[5] Johnson PB, Mazey DJ (1978). Helium gas bubble lattices in face-centred-cubic metals. Nature.

[6] Evans, J. H. Comments on ‘On the onset of void ordering in metals under neutron or heavy-ion irradiation. *Philos. Mag*. **91**, August 2014, 201–203 (2011).

[7] Johnson, P. and Mazey, D. Helium gas-bubble superlattice in copper and nickel. *Nature* **281** (1979).

[8] Johnson PB, Mazey DJ (1995). Gas-bubble superlattice formation in bcc metals. J. Nucl. Mater..

[9] Bolt, H. *et al*. Materials for the plasma-facing components of fusion reactors. In *Journal of Nuclear Materials*. **329–333**(1–3) PART A, 66–73 (2004).

[10] Parish CM, Hijazi H, Meyer HM, Meyer FW (2014). Effect of tungsten crystallographic orientation on He-ion-induced surface morphology changes. Acta Mater..

[11] Tanno T (2009). Effects of transmutation elements on the microstructural evolution and electrical resistivity of neutron-irradiated tungsten. J. Nucl. Mater..

[12] Sikka VK, Moteff J (1972). Superlattice of voids in neutron-irradiated tungsten. J. Appl. Phys..

[13] Tanno T (2007). Effects of Transmutation Elements on Neutron Irradiation Hardening of Tungsten. Mater. Trans..

[14] Krishan K (1982). Invited review article ordering of voids and gas bubbles in radiation environments. Radiat. Eff..

[15] Woo CH, Frank W (1985). A Theory of Void Lattice Formation. J. Nucl. Mater..

[16] Evans JH (1983). Void and bubble lattice formation in molybdenum: A mechanism based on two-dimensional self-interstitial diffusion. J. Nucl. Mater..

[17] Mazey DJ, Eyre BL, Evans JH, Erents SK, McCracken GM (1977). A transmission electron microscopy study of molybdenum irradiated with helium ions. J. Nucl. Mater..

[18] Evans JH (1985). A computer simulation of the two-dimensional SIA diffusion model for void lattice formation. J. Nucl. Mater..

[19] Johnson PB, Mazey DJ, Evans JH (1983). Bubble structures in He+ irradiated metals. Radiat. Eff..

[20] Johnson, P. B. Gas Bubble Lattices in Metals - Fundamental Aspects of Inert Gases in Solids. S. E. Donnelly & J. H. Evans, Eds Boston, MA: Springer US, 167–184 (1991).

[21] Was, G. S. *Fundamentals of Radiation Materials Science*, 1st ed. New York: Springer-Verlag Berlin Heidelberg (2007).

[22] Robinson, A. M. *et al*. The effect of temperature on bubble lattice formation in copper under *in situ* He ion irradiation. *Scr. Mater*., vol. In Press (2017).

[23] Tewary V, Bullough R (1972). Theory of the void lattice in molybdenum. J. Phys. F Met. Phys..

[24] Khachaturyan AG, Airapetyan VM (1974). Spatially periodic distributions of new phase inclusions caused by elastic distortions. Phys. Status Solidi.

[25] Dubinko VI, Slezov VV, Tur AV, Yanovsky VV (1986). The theory of gas bubble lattice. Radiat. Eff..

[26] Amino T, Arakawa K, Mori H (2016). Detection of one-dimensional migration of single self-interstitial atoms in tungsten using high-voltage electron microscopy. Sci. Rep..

[27] Heinisch HL, Singh BN (2002). The effects of one-dimensional migration of self-interstitial clusters on the formation of void lattices. J. Nucl. Mater..

[28] Evans, J. H. Irradiation-Induced Cavity Lattice Formation in Metals. In *Patterns, Defects and Materials Instabilities*, D. Walgraef & N. M. Ghoniem Eds Dordrecht: Springer Netherlands 347–370 (1990).

[29] Evans JH (2007). Comments on the role of 1-D and 2-D self-interstitial atom transport mechanisms in void- and bubble-lattice formation in cubic metals. Philos. Mag. Lett..

[30] Jacques H, Robrock K-H (1981). Elastic after effect studies of molybdenum after electron irradiation at 4.7 K. Le J. Phys. Colloq..

[31] Evans JH (2006). Simulations of the effects of 2-D interstitial diffusion on void lattice formation during irradiation. Philos. Mag..

[32] Nguyen-Manh D, Horsfield AP, Dudarev SL (2006). Self-interstitial atom defects in bcc transition metals: Group-specific trends. Phys. Rev. B.

[33] Derlet PM, Nguyen-Manh D, Dudarev SL (2007). Multiscale modeling of crowdion and vacancy defects in body-centered-cubic transition metals. Phys. Rev. B.

[34] Hähner, P. and Frank, W. A Mesoscopic Theory of Irradiation-Induced Void-Lattice Formation. In *Patterns, Defects and Materials Instabilities* Walgraef, D. & Ghoniem, N. M. Eds Dordrecht: Springer Netherlands, 381–382 (1990).

[35] Sublet J-C, Eastwood JW, Morgan JG (2014). EASY-II Renaissance: n, p, d, α, γ-induced Inventory Code System. Nucl. Data Sheets.

[36] Hasegawa A, Fukuda M, Nogami S, Yabuuchi K (2014). Neutron irradiation effects on tungsten materials. Fusion Eng. Des..

[37] Maury F, Biget M, Vajda P, Lucasson A, Lucasson P (1978). Frenkel pair creation and stage I recovery in W crystals irradiated near threshold. Radiat. Eff. Inc. Plasma Sci. Plasma Technol..

[38] Balluffi RW (1978). Vacancy defect mobilities and binding energies obtained from annealing studies. J. Nucl. Mater..

[39] Ferroni F (2015). High temperature annealing of ion irradiated tungsten. Acta Mater..

[40] Mason DR, Yi X, Kirk MA, Dudarev SL (2014). Elastic trapping of dislocation loops in cascades in ion-irradiated tungsten foils. J. Phys. Condens. Matter.

[41] Yi X, Jenkins ML, Hattar K, Edmondson PD, Roberts SG (2015). Characterisation of radiation damage in W and W-based alloys from 2 MeV self-ion near-bulk implantations. Acta Mater..

[42] El-Atwani O (2014). *In-situ* TEM observation of the response of ultrafine- and nanocrystalline-grained tungsten to extreme irradiation environments. Sci. Rep..

[43] González C, Iglesias R (2014). Migration mechanisms of helium in copper and tungsten. J. Mater. Sci..

[44] Derlet, P. M., Nguyen-Manh, D. and Dudarev, S. L. Multiscale modelling of crowdion and vacancy defects in body-centred cubic transition metals (2007).

[45] Hinks JA, van den Berg JA, Donnelly SE (2011). MIAMI: Microscope and ion accelerator for materials investigations. J. Vac. Sci. Technol. A Vacuum, Surfaces, Film..

[46] Schneider CA, Rasband WS, Eliceiri KW (2012). NIH Image to ImageJ: 25 years of image analysis. Nat Meth.

[47] Ziegler JF (1999). Stopping of energetic light ions in elemental matter. J. Appl. Phys..

[48] Stoller RE (2013). On the use of SRIM for computing radiation damage exposure. Nucl. Instruments Methods Phys. Res. Sect. B Beam Interact. with Mater. Atoms.

[49] A. E521 Standard Practice for Neutron Radiation Damage Simulation by Charged-Particle. *Annu. B. ASTM Stand*. **12.02**, Reapproved, 1–21 (2009).

[50] Rife JC, Donnelly SE, Lucas AA, Gilles JM, Ritsko JJ (1981). Optical Absorption and Electron-Energy-Loss Spectra of Helium Microbubbles in Aluminum. Phys. Rev. Lett..

[51] Donnelly SE (1985). The density and pressure of helium in bubbles in implanted metals: A critical review. Radiat. Eff..

[52] Fréchard S (2009). Study by EELS of helium bubbles in a martensitic steel. J. Nucl. Mater..

[53] Harrison RW, Amari H, Greaves G, Hinks JA, Donnelly SE (2016). Effect of He-appm/DPA ratio on the damage microstructure of tungsten. MRS Adv..

