# Supplementary material for: Engineering self-organising helium bubble lattices in tungsten
Source: Sci Rep. 2017 Aug 10;7:7724. doi: 10.1038/s41598-017-07711-w (PMC5552738; doi:10.1038/s41598-017-07711-w)
Supplement: Supplementary file 1 — Supplementary material [file 41598_2017_7711_MOESM1_ESM.doc]

**Supplementary material for ‘Engineering self-organising helium bubble lattices in tungsten’**

R.W. Harrison*, G. Greaves, J.A. Hinks and S.E. Donnelly

School of Computing and Engineering, University of Huddersfield, Huddersfield, HD1 3DH, UK

Damage and He concentrations were calculated using the *Stopping and Range of Ions in Matter* (SRIM-2013) Monte Carlo computer code [45]. The resultant damage in DPA was calculated using the method proposed by Stoller *et al.* [46] using the ‘Quick’ Kinchin-Pease option of SRIM for 5000 ions with a displacement energy of 90 eV [47] and binding energies set to 0 eV. Helium concentration was determined from SRIM calculations using the ‘Quick’ method to 99,999 ions to ensure good statistics and the foil thickness set to 50 nm. Samples were irradiated to 3.0 DPA (averaged across the TEM foil) using 15, 30, 60 and 85 keV He^+^ ions resulting in implanted He concentrations of 1.2
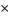
10^5^, 3.0
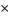
10^4^, 6.5
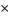
10^3^ and 1.6
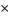
10^3^ appm (averaged across the TEM foil), respectively. SRIM plots of damage (DPA) and helium concentrations in appm of 15, 30, 60 and 85 keV He ions into a 50 nm thick W foil are shown in Figure 1.


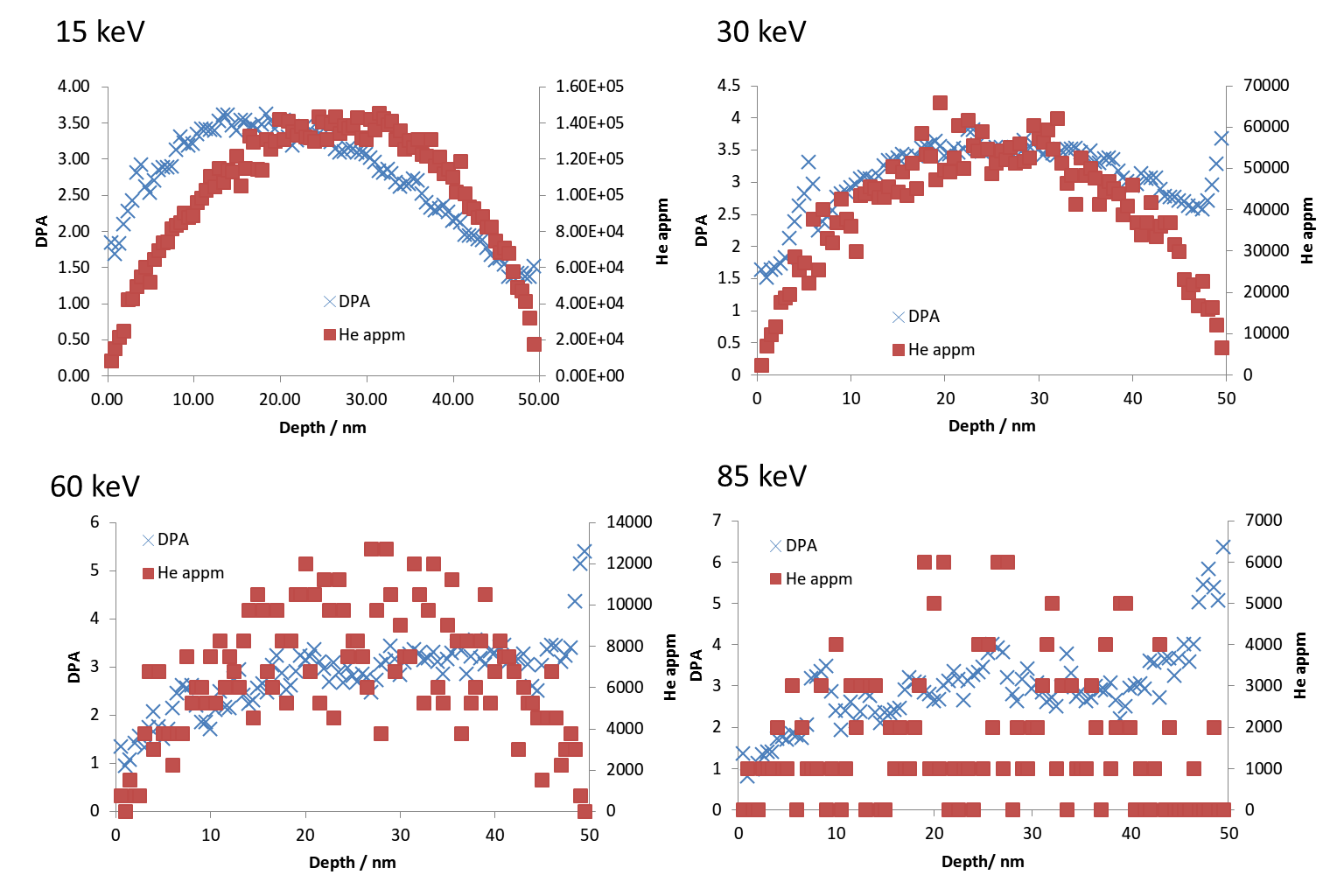


Figure 1. Damage (DPA) and helium concentration (He-appm) plots for 15, 30, 60 and 85 keV He ions into W to a damage dose of 3 DPA

Figure 2, Figure 3 and Figure 4 show TEM images of helium bubble lattices formed under 30, 60 and 85 keV He ion irradiations respectively to 3.0 DPA at 773 K.


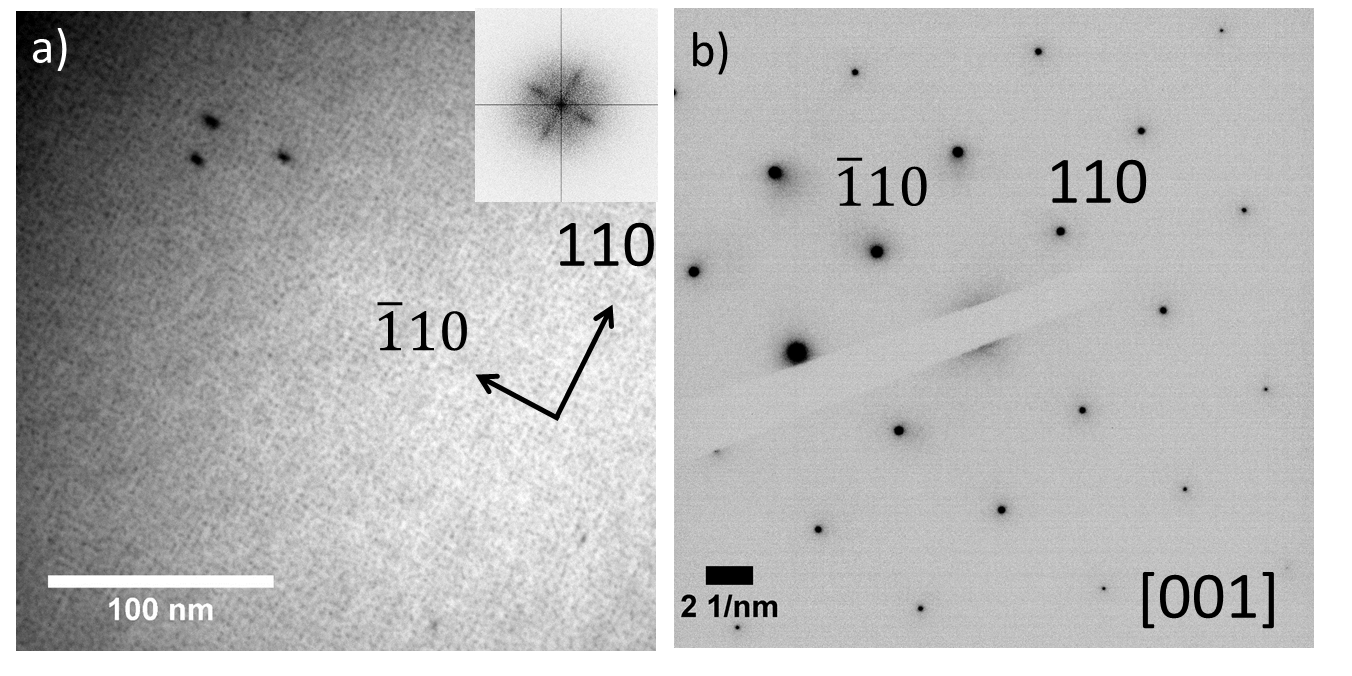


Figure 2. W sample irradiated with 30 keV He ions at 773 K up to 1.5x10^17^ ions.cm^-2^ to 3.0 DPA, a) BF-TEM image of He bubble lattice taken 1.2 μm overfocus, FFT inset shows 4 lobes indicating 3-D ordering, b) SAPD showing crystal is orientated close to [001] zone axis


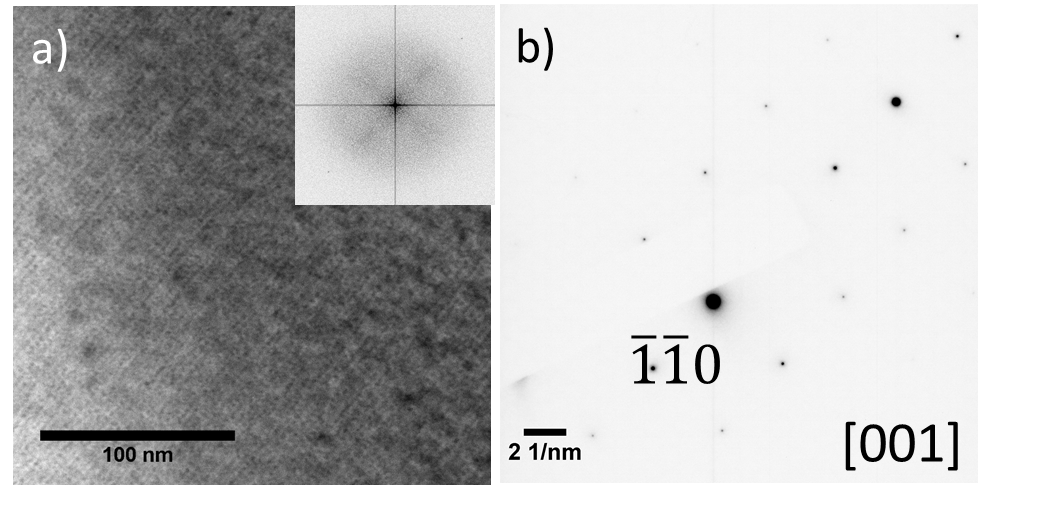


Figure 3. W sample irradiated with 60 keV He ions at 773 K up to 2.4x10^17^ ions.cm^-2^ to 3.0 DPA, a) BF-TEM image of He bubble lattice taken 1.2 μm overfocus, FFT inset shows 4 lobes indicating 3-D ordering, b) SAPD showing crystal is orientated close to [001] zone axis


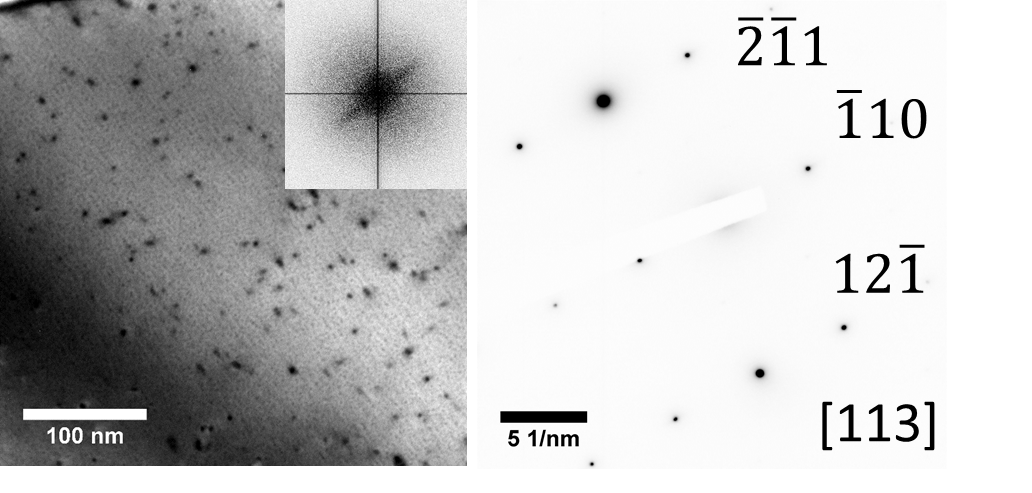


Figure 4. W sample irradiated with 85 keV He ions at 773 K up to 3.2x10^17^ ions.cm^-2^ to 3.0 DPA, a) BF-TEM image of He bubble lattice taken 1.2 μm overfocus, FFT inset shows ordering in {110} planes b) SAPD showing crystal is orientated close to [113] zone axis.
